# Feasibility of measuring urethral pressure during female midurethral slings: Case report

**DOI:** 10.1097/MD.0000000000021100

**Published:** 2020-07-10

**Authors:** Ling-Feng Meng, Miao Wang, Wei Zhang, Xiao-Dong Liu, Yao-Guang Zhang

**Affiliations:** Department of Urology, Beijing Hospital, National Center of Gerontology, Institute of Geriatric Medicine, Chinese Academy of Medical Sciences, Beijing, China.

**Keywords:** case report, intraoperative, midurethral slings, stress urinary incontinence, urethral pressure profilometry

## Abstract

**Rationale:**

Stress urinary incontinence (SUI) refers to the involuntary leakage of urine when abdominal pressure increases. Midurethral slings (MUS) have become the main surgical method for treating SUI, but no quantitative standard for the degree of sling tightness during operation exists. We achieved this quantitative measurement using ambulatory urodynamic equipment.

**Patient concerns:**

A 49-year-old woman presented to our hospital with intermittent urine leakage. Five pads were used daily to keep the vulva dry. The preoperative urethral pressure profilometry (UPP) showed that maximum urethral pressure (MUP) was 54 cmH2O and maximum urethral closure pressure (MUCP) was 53 cmH2O.

**Diagnosis:**

According to the medical history and examination findings, the patient was diagnosed as SUI.

**Interventions:**

The MUS and UPP were performed.

**Outcomes:**

The intraoperative UPP showed that MUP was 29 cmH2O and MUCP was 17 cmH2O. Three months after the operation, the patient was followed up by telephone. The amount of urine pad usage decreased from 5 pads/d to 0 pads/d, reaching the social control standard (0–1 pads/d). The patient's international consultation on incontinence questionnaire short form score decreased from 18 to 5, and their incontinence quality of life score increased from 12.5 to 78.4. The effect of urine control was satisfactory, and no complications occurred.

Five months after operation, the patient was reexamined in the outpatient department. The UPP showed that the MUP was 98 cmH2O and the MUCP was 72 cmH2O. The patient still uses 1 pad/day. The international consultation on incontinence questionnaire short form score is 6 and incontinence quality of life score is 79.5. The curative effect is stable.

**Lessons:**

MUS has become an effective surgical method for SUI, and the tightness of the sling directly affects the surgical outcome. We have achieved the measurement of urethral pressure during MUS. However, although we found that there is no obvious clinical significance of urethral pressure measurement in MUS operation, future research will benefit from our findings by improving upon our study design to help standardize the clinical diagnosis and treatment of MUS.

## 1 Introduction

Stress urinary incontinence (SUI) refers to the involuntary leakage of urine when the abdominal pressure increases, such as while sneezing or coughing. It is reported that the incidence of female SUI varies from 15% to 49% and increases with age.^[1–3]^ Although there is no direct relationship between SUI and life expectancy, its pathological changes have significant effects on the physical health, quality of life, and psychological and emotional state of patients. Nearly a quarter of women with incontinence complain that they cannot work properly because of an involuntary leak.

In recent years, the American Urological Association has updated the guidelines for the treatment of female SUI. The updated guidelines still recommend the use of midurethral slings (MUS) as an effective method for the treatment of female SUI (Evidence Level: Grade A; Strongly recommended).^[4]^ The main purpose of patients receiving MUS is to achieve satisfactory control of urine, and the increase of urethral resistance after implantation is considered to be an important factor to achieve the control of urine. At present, it is believed that the key to control urine after MUS is to tighten the appropriate sling; however, there have been few investigations evaluating the pressure of the sling on the urethra. Further, urethral pressure profilometry (UPP) is not part of the routine diagnosis, treatment, and follow-up of SUI patients.

In the present study, we review the diagnosis and treatment of a female SUI patient admitted in July 2019, and we introduce the measurement method and results of UPP during MUS.

## 2 Case presentation

A 49-year-old Chinese woman was admitted to our hospital complaining of intermittent leakage of urine for 15 years, aggravated severely within the last 2 years. The patient had urinary leakage after strenuous activity 15 years ago and was not treated. In the past 2 years, the symptoms have been aggravated dramatically, and urine leakage occurs during activities. There is no leakage of urine while sitting or lying down. At presentation, the patient was using 5 pads/d to control the leakage of urine. The patient's past history of illness included a total hysterectomy performed in the local hospital in 2002 due to severe hyperplasia of cervical epithelium. The patient denied having any other diseases or allergies. Further, no smoking or drinking history was conveyed, and there was no family genetic history.

Physical examination upon admission showed a 20-cm long transverse scar can be seen on the pubic symphysis. A bladder neck lifting test was positive, and the swab test was also positive. Laboratory findings were unremarkable. A routine preoperative examination, urodynamics, and UPP was performed (Laborie Delphis, Laborie Medical Technologies Canada unlimited liability corporation, 7Fr T-DOC air-charged dual sensor catheter, traction device pull-out the pressure pipe at a speed of 1 mm/s).

**Figure 1:**
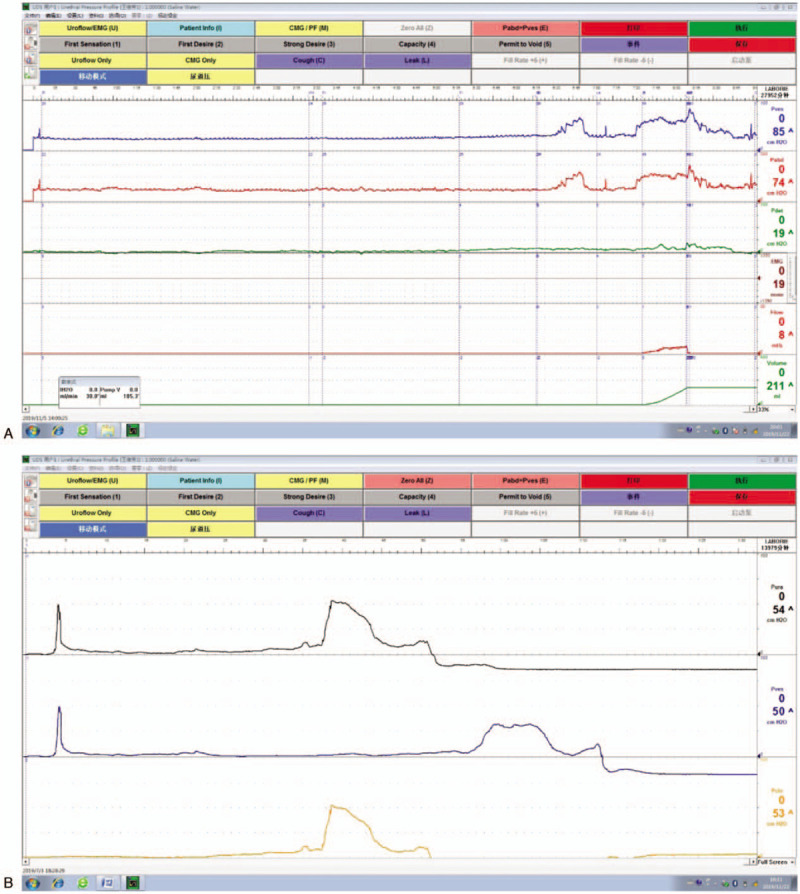
Preoperative examination. (A) Urodynamics showed that: 1. Bladder function and compliance were normal; 2. Detrusor contractile force decreased to 19 cmH2O; 3. VLPP was 65.7 cmH2O, CLPP was 45 cmH2O; (B) Preoperative UPP showed that the MUP was 54 cmH2O and MUCP was 53 cmH2O. CLPP = cough-induced leak point pressure, MUP = maximum urethral pressure, UPP = urethral pressure profilometry, VLPP = Valsalva leak point pressure.

No obvious abnormalities were found in the routine examination. The results of urodynamics and UPP showed that bladder function and compliance were normal; detrusor contractile force decreased to 19 cmH2O; Valsalva leak point pressure was 65.7 cmH2O and cough-induced leak point pressures was 45 cmH2O (Fig. 1A); and MUP was 54 cmH2O and MUCP was 53 cmH2O (Fig. 1B).

**Figure 2:**
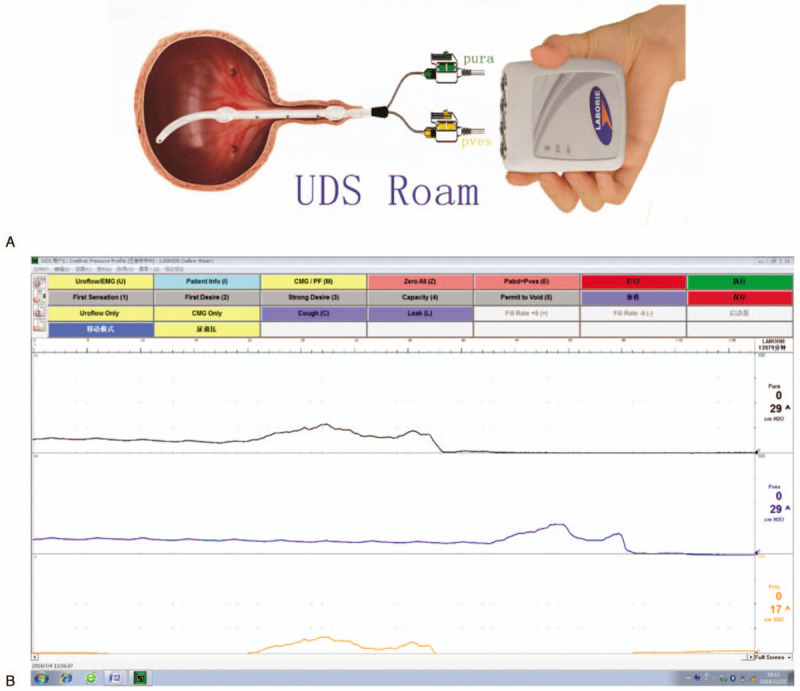
Intraoperative UPP. (A) Schematic diagram of intraoperative UPP; (B) During operation, the MUP was 29 cmH2O and MUCP was 17 cmH2O. MUCP = maximum urethral closure pressure, MUP = maximum urethral pressure, UPP = urethral pressure profilometry.

The final diagnosis of the presented case was SUI. The transobturator vaginal tape inside-out technique was performed in July 2019. After the sling was placed, the ambulatory urodynamic device (Laborie, Laborie Medical Technologies Canada unlimited liability corporation) was used with a 7Fr T-DOC air-charged catheter for intraoperative UPP (Fig. 2A). The results showed that MUP was 29 cmH2O and MUCP was 17 cmH2O (Fig. 2B).

Three months after the operation, the patient was followed up via telephone. Urinary pad usage was reduced from 5 pads/d to 0 pads/d, the international consultation on incontinence questionnaire short form score was reduced from 18 to 5, the incontinence quality of life score had increased from 12.5 to 78.4, and the effect of urine control was satisfactory.

**Figure 3:**
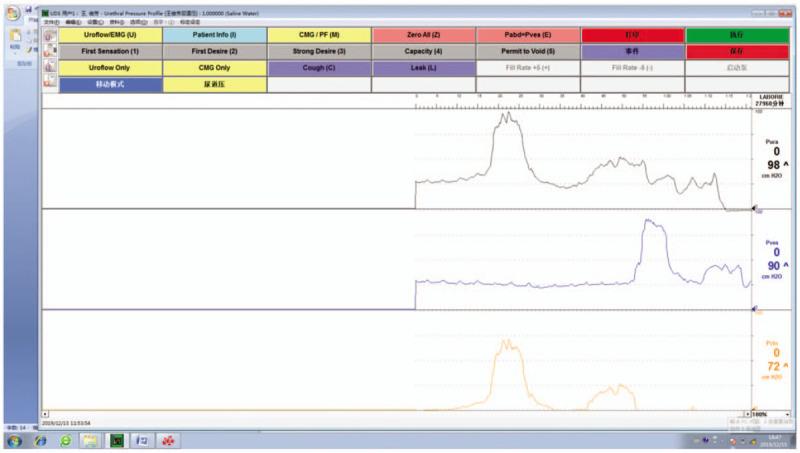
UPP at 5 mo after operation. After 5 mo of operation, the MUP was 98 cmH2O and MUCP was 72 cmH2O. MUCP = maximum urethral closure pressure, UPP = urethral pressure profilometry, MUP = maximum urethral pressure.

Five months after operation, the patient was reexamined in the outpatient department and underwent UPP. The results showed that MUP was 98 cmH2O and MUCP was 72 cmH2O (Fig. 3). The patient still uses 1 pad/d. The incontinence questionnaire short form score is 6 and incontinence quality of life score is 79.5. The curative effect is stable.

The patient provided informed consent. The study design was approved by the appropriate ethics review board and was CARE compliant.

## 3 Discussion

The purpose of the MUS operation is to obtain urinary control, to achieve the standard of complete urinary control or social urinary control, and to minimize the occurrence of complications. Therefore, it is of great significance to evaluate the changes of urinary pressure in patients undergoing MUS operations.

According to the International Continence Society's report on the standardization of UPP, the clinical application of UPP is still unclear, but there is no doubt that UPP is of great significance to the urinary control mechanism.^[5]^

However, only a small number of studies have evaluated the distribution of preoperative urethral pressure in women with SUI. Further, no study has explored urethral pressure during MUS operations.

Kuralay Sharipova et al^[6]^ included 130 patients with urinary incontinence (including 75 patients with simple SUI and 55 patients with simple urgency urinary incontinence). The MUCP of patients with simple SUI was 61.0 (43.5–78.5) cmH2O, significantly lower than 66.0 (52.0–96.0) cmH2O of patients with simple urgency urinary incontinence. This is consistent with the results of our preoperative examination.

We have previously reported and confirmed the significance of UPP in the operation of artificial urinary sphincter implantation, and pointed out that this method is also applicable to female MUS operation.^[7]^

However, we found that in this case, the preoperative MUP was 54 cmH2O and the MUCP was 53 cmH2O, while the intraoperative MUP was 29 cmH2O and the MUCP was 17 cmH2O, which was significantly lower than that before operation.

This finding is quite different from our previous finding in artificial urinary sphincter implantation. In another patient who received transobturator vaginal tape inside-out, we performed an UPP examination during the operation. The results were similar. Both MUP and MUCP were significantly lower than those prior to the operation.

We speculate that the reason for this is that, in order to be able to locate the urethra more clearly, the operators often insert 16Fr catheters before the operation. After the sling was placed, we performed intraoperative UPP to assess the pressure of the urethra.

At this time, we needed to remove the 16Fr catheter and insert the 7Fr air-charged catheter while the urethra was expanded to some extent by the 16Fr catheter.

Therefore, compared with the dilated urethra, the detection efficiency of the 7Fr air-charged catheter in the measurement of urethral pressure is significantly reduced.

Although the method of UPP has been standardized, as far as we know, there is no recognized normal or reference value. According to Chinese experts, the average MUP of normal women aged 45 to 64 in China is 74 (40–100) cmH2O, and the reference range of MUCP is 70 to 90 cmH2O.^[8]^ The patient's results of UPP showed that the MUP was 98 cmH2O and the MUCP was 72 cmH2O, all of which were within the normal range, and the curative effect was stable. The results of the above data confirm the efficacy of MUS in SUI, and it is confirmed by UPP that it is to help patients achieve satisfactory control of urine by increasing urinary resistance.

Previously, the reason for female SUI was explored by Delaney Jo.^[9]^ It was found that MUCP was the most significant difference between the patients with SUI and the asymptomatic population. MUCP in the Sui group was 43% lower than that in the asymptomatic group. This is consistent with our conjecture and research results.

However, in the current study, due to the limitations of the operation and equipment, it seems that there is no obvious clinical significance in the measurement of urethral pressure in women's MUS operation. The UPP after MUS operation showed that the patient's MUP and MUCP reached the normal range and achieved satisfactory control of urine. The short-term follow-up effect was stable.

This finding suggests that we should further evaluate the feasibility of UPP in female MUS and to avoid unfounded intraoperative UPP examination. Because the T-DOC air-charged catheters are disposable medical consumables, their unnecessary overuse will further increase medical costs and waste medical resources. Therefore, we look forward to future research that will expand upon our methods to further improve the method of detecting urethral pressure in women's MUS operations, helping to standardize its clinical diagnosis and treatment.

In order to understand the relationship between the range of intraoperative urethral pressure and the effect of urinary control, we have achieved the measurement of urethral pressure in women's MUS operations. However, limitations in the operation and equipment of our study have indicated that there is no obvious clinical significance in the measurement of urethral pressure in MUS. Therefore, it is necessary to further evaluate the value of UPP in MUS.

## Author contributions

**Data curation:** Miao Wang, Xiao-Dong Liu

**Project administration:** Yao-Guang Zhang

**Supervision:** Yao-Guang Zhang

**Writing – original draft:** Ling-Feng Meng, Miao Wang

**Writing – review & editing:** Wei Zhang, Yao-Guang Zhang
